# Brain-machine interfaces as a challenge to the “moment of singularity”

**DOI:** 10.3389/fnsys.2014.00213

**Published:** 2014-12-17

**Authors:** Philip Kennedy

**Affiliations:** Human Recording Lab, Neural Signals Inc.Duluth, GA, USA

**Keywords:** brain interfacing, brain augmentation, hybrid human robot, brain stimulation, brain recording, artificial intelligence

Ray Kurzweil predicts that artificial intelligence will equal and then surpass human intelligence in the not-too-distant future, in what he calls the “moment of singularity.” Advances in brain/machine interfacing (BMI) may be viewed as a challenge to this futuristic prediction. BMIs strive to instrument human brains with unlimited memory, calculation, and communication abilities, which provide a competitive edge to human brain power versus artificial intelligence. This paper makes a case for a hybrid human/robot that merges the brain function with artificial intelligence components, and prevents the “moment of singularity” from ever occurring.

## Kurzweil predictions

Kurzweil has made predictions regarding computers and artificial intelligence in his four books (Kurzweil, 1992, 1999, 2005, 2012). In his 2005 book “The Singularity is Near, when Humans transcend Biology” (Kurzweil, 2005), he predicts that by the year 2045 artificial intelligence will be equal to human intelligence, an event he calls the “moment of singularity.” His most recent book “How to create a Mind” (Kurzweil, 2012) brings the singularity date forward in time to 2029. He argues persuasively in that book that artificial intelligence embodied in computers will far surpass human intelligence as the 2030s roll on. He makes an excellent case throughout the text of how the brain operates as a hierarchical system and how this is important in how to create a mind.

In that book (Kurzweil, 2012), he makes a second somewhat contentious point about how the neo-cortex can be enhanced by artificial intelligence. He suggests on page 244 that using intelligent computers no bigger than a red blood cell, intelligence will be introduced into the biological brain in a minimally invasive way via the blood stream. Thus, rather than just making the case for artificial intelligence within a computer, he alludes to the scenario wherein we will enhance our present brains. However, he does not say *when* it will happen in a meaningful way. He does state that artificial intelligence will equal human intelligence by 2029 (the moment of singularity), he does not predict when intelligence will enhance biology brains. Perhaps detailing “how and when” may well be the subject of his next book.

## My own futuristic view

This issue of “how and when” has already been proposed in my book: “2051” (Royal, 2013). This brief novelette predicts via a human story that brain machine interfacing will provide humans with a robotic shell containing the brain, and with all emotional and intellectual functions enhanced by information accessible from within the robotic shell. This human brain/robot allow the loving couple to explore the universe. The hypothesis is that enhanced brains will be incorporated into robotic machines that will *not* lose human status. Clearly, success in this venture will delay the original singularity moment of 2045, though perhaps not Kurzweil's revised date of 2029. No mention is made in my novel of the 2029 date, being unknown at the time of writing in 2006 and 2007 (Royal, 2013). (Full disclosure: This writer published “2051” under a pseudonym, Alpha O. Royal).

## Brain-machine interfaces

Interestingly, many members of the brain-machine/computer interfacing research community believe that BMIs may have a key role in the advancement of intelligence that constitutes the core of Ray Kurzweil's hypotheses. The challenge is to delay or perhaps completely abort his prediction. This could be achieved by enhancing the human brain so that we stay one step ahead of intelligent computers. Think: If it were possible to provide humans with instant and total memory, access to all information, infinite calculation ability and instant communication with whomever, whenever and wherever, we could have intelligence that would be superior to any present day intelligence that a computer has today (Li et al., 2012). Enhanced memory and knowledge can provide an individual with an unparalleled asset and make you superior to all other humans except those others who have this asset. Having all calculation abilities and instant communication would complete the superior human being (Fitzsimmons et al., 2007; O'Doherty et al., 2011). But would we be superior to Kurzweil's predicted intelligent computers? Is it possible that before 2045, the original year of singularity, or 2029, we humans could have superior intelligence and thus delay or abort Kurzweil's prediction?

## The alternative hypothesis

The hypothesis in “2051” is based on our present technology and where it is likely heading. For example, if recording and stimulating electrodes continue on their present developmental path, it is likely that the brain can be instrumented completely (Marblestone et al., 2013). Recording and stimulating electrodes would be placed over the hemispheric cortices primarily, and also within deeper structures such as the basal ganglia as is done today for Parkinson's disease (Marblestone et al., 2013). These recording and stimulating electrodes would provide all essential inputs and outputs.

## Issues with this hypothesis

A very important problem is immediately raised however: How can the instrumented brain assimilate all the information that would descend on it? How could we restrict and channel it to avoid being overwhelmed with useless information (Fitzsimmons et al., 2007; O'Doherty et al., 2011)? In these papers, monkeys received artificial sensations via intra-cortical stimulation. Initially, the brain was overwhelmed and the animals did not understand the meaning of this input. However, later they started to make sense of the artificial sensation, that is they developed a new sense. They then learned new discrimination tasks faster. Carefully placed inputs would help. For instance, visual input would travel from artificial eyes to the electrodes in the visual cortex, and auditory input would travel to the auditory cortex, and so on (Fagg et al., 2007; Bensmaia and Miller, 2014). But when downloading information does it consciously or subconsciously arrive at visual or auditory cortex? Would it be better if the information goes directly to the hippocampus, originally thought to be the “gateway to memory,” or to other sites? If through the hippocampus, is the information available to consciousness or is it only conscious and available on demand when needed? If that is how it is to occur, then why would the information not overload memory storage in the brain? How would our biological brain be capable of storing all knowledge? A more likely storage alternative is that all knowledge would be stored in attached computers or “in the cloud” to be available when needed (Li et al., 2012). As Kurzweil predicts, all the information is to be available within the cloud (Kurzweil, 2012). If the cloud is the modus operandi, then how would we access the information? Perhaps the same way we search and access information using Google or other search engines. These are the type of problems that we cannot answer with our present stage of knowledge of the brain. These issues are the important ones. Only by understanding the neurophysiology of the brain more fully, can we approach an answer to these questions.

## Other challenges

Other challenges include how the instrumented brain will interact with the body? How will it comport with the body? If the brain is thoroughly instrumented with electrodes does it even need the body? After all, the body will age and become diseased with the usual cancer, cardiovascular, or other problems. A solution is to replace the aging or diseased body and support the brain by other means (provided of course the brain itself is not diseased). Basically, all the brain requires to remain functional is a blood and cerebrospinal fluid supply that provides oxygen and nutrients, and removes metabolic products, while inputs and outputs are provided by instrumenting the brain. Thus, metabolic requirements could be provided, not by our natural bodies, but by artificial means such as miniature heart/lung/nutrient machines or internal factories. Nanotechnology is needed to provide miniaturization of hardware components in such a device and will require external replenishment of supplies from time to time or an indwelling biological factory to provide these essential ingredients.

## Hybrid/human robot

**Figure 1:**
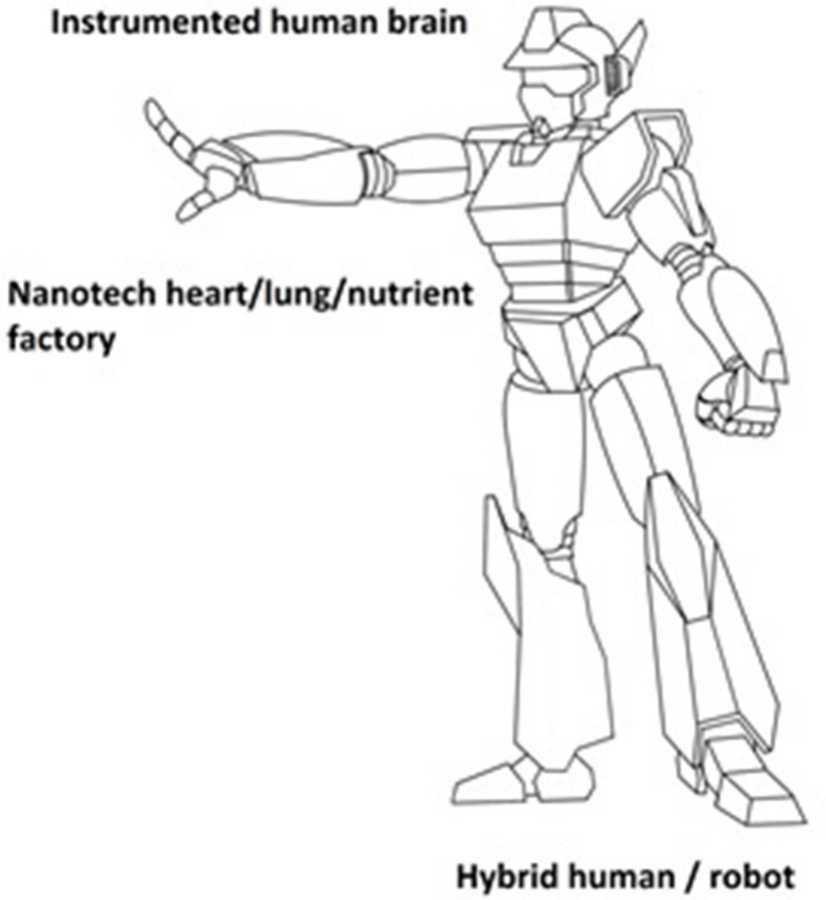
**Theoretical hybrid human / robot**.

If an artificial heart/lung/nutrient factory were to be feasible, then why would we need a body? Well, we wouldn't. There would be no need for a biological body to provide mobility. Mobility and other functions could be maintained by a robotic shell that contains the heart/lung/nutrient factory that are all controlled by the instrumented brain. Advances with robotic hardware, software, and nanotechnology make such a development a strong possibility. It appears likely that we could end up with a *hybrid robot containing an instrumented human biological brain that controls the robotic body* (see Figure 1). This is a different outcome than Kurzweil's prediction of artificial intelligence in a computer, or humans with enhanced brains that have received artificial intelligence via red blood cells.

Clearly, there is a big problem with this scenario. That is, how to maintain a supply of human brains? If all the bodies are gone, where do the ova and sperm come from to create more humans with brains? Banked sperm and ova are a possibility. They would be fertilized as needed and developed into humans by baby factories. Ugh! Without a supply of human brains the robots would take over completely. In that scenario, Kurzweil's prediction will only be delayed as long as there are human brains available. Once the human brains have all died off, Kurzweil's prediction will come true. *Oh darn, you think, he's right again*. Ah, but wait, tissue can be regrown. Perhaps a new human body and brain can be regrown completely and maintained, if not young, at least virile (Monaghan and Maden, 2013; Racine et al., 2014). In that case, human reproduction can take place in a biological fashion, without baby factories, thus maintaining the supply of human brains.

## The ethical issues

Then there are the ethical issues (Albrecht and Devlieger, 1999; Amundson, 2010; Glannon, 2014). If (a) enhanced humans, (b) computers with artificial intelligence or (c) hybrid human/robots, are fully realized, then these beings would be superior to all others who are not yet enhanced. Clearly, this would create a group of superior beings that governments would want for their own use as part of their defense/offense capabilities to protect against international aggression and threats. These hybrids could be also used as national police. This bears on the very serious issue of control of the native population, a control issue that is becoming ever more prevalent these years. The present ability to know what humans think and do through access to their communications has provided governments with knowledge that could lead to total domination of the native population. Such domination could, and likely would, be enforced by the hybrid human/robots. I raise this issue because scientific knowledge and technological achievements do not occur in a vacuum. They always have societal implications and no implication is more serious than this potential technology. *Over half a century ago*, President Eisenhower warned about these dangers in his farewell speech to the nation 3 days before he left office in 1960 as shown in a news reel of his address (Webster, 2014). Of course he made no specific mention of the technological advances that are occurring today. He discussed his fear that the military/industrial complex would become “the whole dog rather than just the teeth.” The answer to this ethical dilemma is simply that the hybrid human/robot should be available to all people and not restricted to any one group, whether it be government or otherwise.

## Conclusions

So the serious challenges for the brain computer interface community are not just technical, they are ethical as well. Perhaps we should not go there, but technology always takes on a momentum of its own in its inevitable and unrelenting march forwards. We cannot flee the challenge of delaying or avoiding Kurzweil's prediction. We must embrace it. That is our responsibility. But let's tread lightly and very, very carefully.

### Conflict of interest statement

I wrote the book under a pseudonym: Royal A.O. 2051. Amazon Digital Services.
